# Herlyn-Werner-Wunderlich Syndrome Presenting as an Incidental Retrovesical Cyst in an Infant: A Case Report

**DOI:** 10.7759/cureus.78190

**Published:** 2025-01-29

**Authors:** Ammar Khamis, Fatema Kadhem

**Affiliations:** 1 Radiology, Salmaniya Medical Complex, Manama, BHR

**Keywords:** herlyn-werner-wunderlich syndrome, müllerian duct anomalies, obstructed hemivagina, renal agenesis, retrovesical cyst, uterovaginal septum, vaginoplasty

## Abstract

Müllerian (paramesonephric) ducts are the primordial components of the female reproductive tract, and defects in their development can lead to a range of uterine anomalies, including hypoplasia, agenesis, and other structural abnormalities. Herlyn-Werner-Wunderlich (HWW) syndrome is a rare variant of Müllerian duct anomalies characterized by uterine didelphys, obstructed hemivagina, and ipsilateral renal agenesis. This syndrome is often diagnosed incidentally, typically presenting with symptoms related to urinary tract or reproductive system malformations. Early recognition is crucial to preventing complications such as infertility, chronic pain, and renal dysfunction. This case describes a six-month-old female infant, a product of in vitro fertilization, who was admitted to the neonatal intensive care unit due to persistent abdominal distention and vomiting. Initial investigations for Hirschsprung’s disease were negative, and radiologic findings revealed a single kidney and grade III vesicoureteric reflux. At six months of age, the infant was readmitted with fever and recurrent vomiting. Imaging studies, including ultrasound and CT, revealed a large retrovesical cyst and left renal agenesis. An MRI demonstrated that the cyst arose from the uterus and vagina, likely due to a uterovaginal septum. Surgical intervention included vaginotomy and vaginoplasty, successfully resolving the cyst and restoring vaginal patency. Postoperative imaging confirmed an empty uterine cavity and no further cyst formation. This case underscores the importance of understanding the embryological development of the female reproductive and renal systems, as well as the early clinical suspicion of HWW syndrome. Timely diagnosis and intervention are critical to preventing complications such as obstructed menstruation, pelvic pain, and renal dysfunction. Despite its rarity, early recognition of this syndrome in infants and young children can significantly improve patient outcomes and quality of life.

## Introduction

Müllerian (paramesonephric) ducts are the primordial components of the female reproductive tract, and defects in their development can lead to a range of uterine anomalies, including hypoplasia, agenesis, and other structural abnormalities. Herlyn-Werner-Wunderlich (HWW) syndrome is a rare variant of Müllerian duct anomalies characterized by the combination of uterine didelphys, obstructed hemivagina, and ipsilateral renal agenesis [1-3]. This syndrome is typically diagnosed in females, often presenting with a variety of symptoms related to urinary tract or reproductive system malformations. The condition was first described in 1971 by Herlyn and Werner, later expanded by Wunderlich, and has since been recognized as a distinct syndrome with significant implications for affected individuals [2,4].

The clinical presentation of HWW syndrome can vary widely, but it is often diagnosed incidentally when imaging studies reveal a retrovesical cyst or pelvic mass in the context of urinary tract symptoms, such as abdominal distention or recurrent urinary tract infections. The combination of a unicornuate uterus with a nonfunctioning rudimentary horn and a nonpatent hemivagina can lead to complications such as cyst formation due to obstructed menstrual flow or associated renal anomalies [2-4]. Though it is a rare condition, early recognition of HWW syndrome is important for preventing long-term complications, including infertility, chronic pain, and renal dysfunction. Management of the syndrome typically involves a multidisciplinary approach with attention to both the urological and gynecological anomalies, often requiring surgical intervention to correct the structural abnormalities and alleviate symptoms [1,5].

## Case presentation

A six-month-old female infant, a product of in vitro fertilization, was born at term following an uncomplicated normal vaginal delivery. The infant’s birth weight was appropriate for gestational age, and she did not experience any immediate complications during the perinatal period.

The infant was admitted to the neonatal intensive care unit due to persistent abdominal distention and vomiting. A thorough workup was initiated, including investigations for Hirschsprung’s disease, which were ultimately negative. Radiologic findings during this hospitalization revealed a single kidney and grade III vesicoureteric reflux. An MRI of the brain was performed and showed no abnormalities. Following stabilization, the patient was discharged with follow-up recommendations to monitor her urological concerns.

At six months of age, the patient was readmitted through the pediatric emergency department with fever, recurrent vomiting, and irritability. The vomiting was nonbilious, and there were no associated symptoms of abdominal pain or diarrhea. Physical examination revealed a moderately dehydrated infant with normal vital signs and no significant abdominal tenderness. A cleft palate was noted, but no other findings were present to suggest a primary gastrointestinal or infectious etiology.

**Figure 1:**
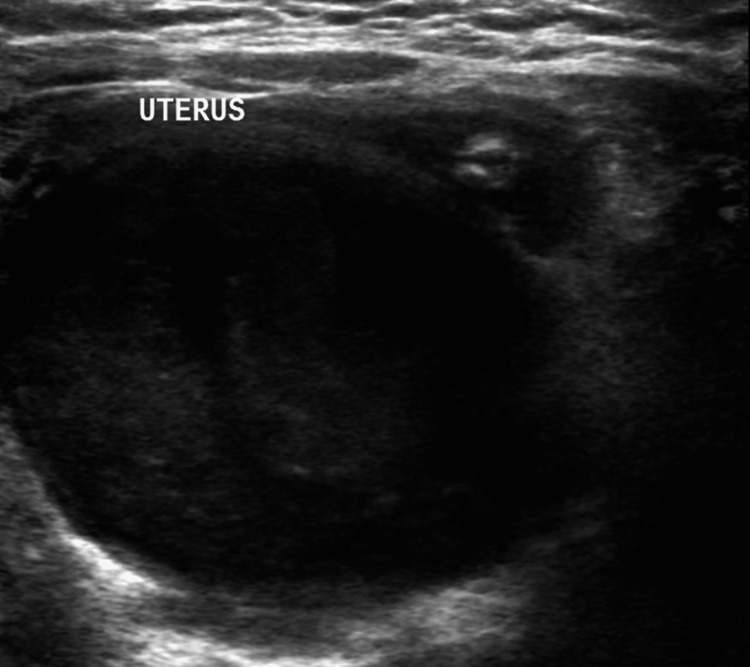
Grayscale ultrasound of the abdomen and pelvis showing a large retrovesical cyst containing turbid fluid.

**Figure 2:**
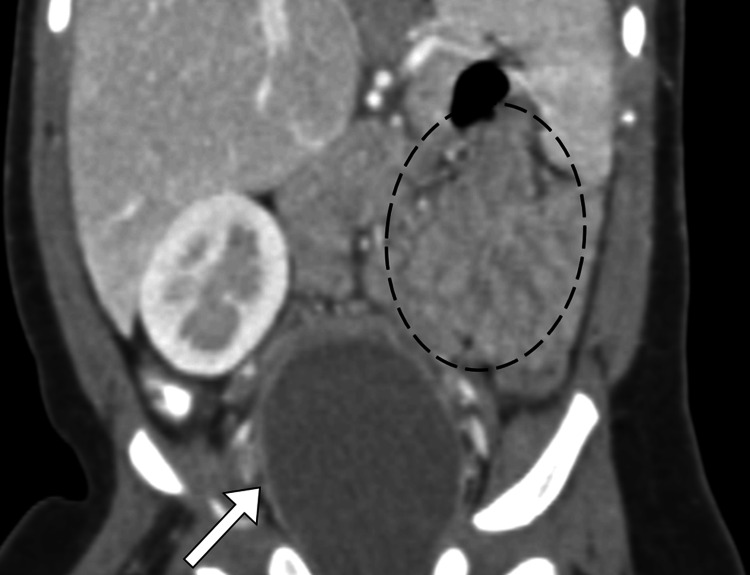
Coronal contrast-enhanced computed tomography image demonstrating a large retrovesical abdominopelvic cyst (arrow). The cyst is clearly visible in the pelvic region, with the additional finding of left renal agenesis (encircled).

Given her prior history of gastrointestinal symptoms, intussusception was suspected, and an abdominal ultrasound was performed. The ultrasound did not show evidence of intussusception but did reveal a large retrovesical cyst (Figure 1). A subsequent abdominopelvic CT scan confirmed the presence of this cyst, which contained turbid fluid, along with the finding of left renal agenesis (Figure 2).

**Figure 3:**
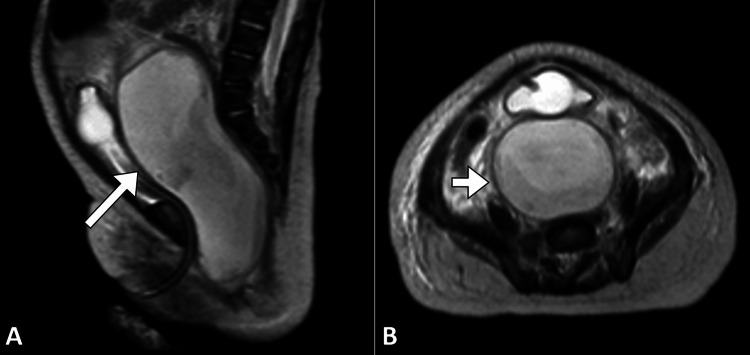
MRI abdominopelvic sagittal (A) and axial (B) T2-weighted images showing a distended uterus and vagina filled with fluid (arrow), consistent with an obstructed hemivagina.

An urgent preoperative MRI was conducted, which demonstrated a large retrovesical cyst extending into the abdominal cavity. The cyst was found to arise from the uterus and vagina, most likely due to the presence of a uterovaginal septum. Left renal agenesis was again noted on the MRI. These findings indicated a complex congenital malformation involving the lower urinary and reproductive systems (Figure 3).

The patient was taken to the operating room for surgical evaluation. Examination under anesthesia revealed normal external genitalia, including the labia and clitoris, though a large pelvic mass was noted, bulging out from the vaginal opening. Intraoperatively, the patient was found to have distal vaginal atresia, which was surgically corrected through vaginotomy. The cystic fluid was completely evacuated, and vaginoplasty was performed to reconstruct the vaginal opening.

Postoperative imaging, including an ultrasound, showed an enlarged uterus with an empty endometrial cavity. The previously noted large retrovesical cyst had resolved, and the vaginal opening was patent. The patient was closely monitored in the postoperative period for any signs of infection or further complications.

## Discussion

HWW syndrome is a rare congenital disorder resulting from a malformation of the Müllerian ducts during embryonic development. It is characterized by a combination of uterine didelphys, obstructed hemivagina, and ipsilateral renal agenesis [2,3]. While the condition is uncommon, its clinical significance lies in the potential for misdiagnosis, as the symptoms often overlap with more prevalent disorders [1-4]. In the case of our patient, the incidental finding of a large retrovesical cyst during imaging raised suspicion for HWW syndrome, highlighting the importance of thorough evaluation when encountering unexplained abdominal or pelvic masses in female infants. Early identification of this syndrome is crucial, as the obstructed hemivagina may lead to significant complications such as hematocolpos, pelvic pain, and recurrent infections, while the ipsilateral renal agenesis can result in long-term renal compromise if not monitored appropriately [2,6].

The clinical presentation of HWW syndrome can be variable, ranging from asymptomatic cases to those presenting with urinary or reproductive tract symptoms [1-4]. In this case, the child’s persistent abdominal distention and vomiting were initially misattributed to common gastrointestinal conditions such as Hirschsprung’s disease, underscoring the challenge in diagnosing rare syndromes with nonspecific symptoms. The use of radiologic imaging, particularly ultrasound, CT, and MRI, is pivotal in diagnosing HWW syndrome. Imaging studies in our patient demonstrated a large retrovesical cyst, which was later confirmed to arise from the uterine and vaginal structures, further supporting the diagnosis of an obstructed hemivagina secondary to the uterovaginal septum [5,6]. This case emphasizes the value of comprehensive imaging, which not only helps in diagnosing rare anomalies but also aids in preoperative planning for surgical correction.

Management of HWW syndrome often involves a multidisciplinary approach, including urology, gynecology, and sometimes nephrology, due to the associated renal anomalies [5-8]. Surgical intervention is typically required to relieve any obstructive symptoms, correct the vaginal atresia, and address the presence of the nonfunctional rudimentary uterine horn [1,7]. In this case, the child underwent successful surgery involving vaginotomy and vaginoplasty, which resolved the retrovesical cyst and restored normal vaginal patency. Postoperative follow-up imaging confirmed the absence of further cyst formation and revealed a structurally normal, albeit empty, uterine cavity. However, the child will require long-term monitoring to assess renal function, given the presence of ipsilateral renal agenesis, and for possible recurrence of obstructive symptoms related to the vaginal septum.

Although the prognosis for HWW syndrome varies, early diagnosis and surgical management can significantly improve the quality of life for affected individuals [1-4]. Long-term follow-up is necessary to monitor for potential complications such as renal dysfunction, infertility, and chronic pelvic pain. Moreover, given the rarity of this syndrome, this case underscores the importance of maintaining a high index of suspicion when evaluating infants with atypical urogenital or abdominal findings. Multidisciplinary collaboration is essential in providing comprehensive care and ensuring optimal outcomes for patients with this complex condition.

## Conclusions

This case highlights the critical importance of understanding the embryological development of the female reproductive and renal systems, as well as the interplay between them. Early clinical suspicion and awareness of HWW syndrome are essential for timely diagnosis and intervention, preventing long-term complications such as obstructed menstruation, pelvic pain, and renal dysfunction. Given the rarity of the condition, it is often missed or diagnosed late, typically at menarche, underscoring the need for heightened vigilance in identifying this syndrome in infants and young children. Early recognition and appropriate management can significantly improve outcomes and quality of life for affected individuals.
